# Supplementary material for: Oocytes can repair DNA damage during meiosis via a microtubule-dependent recruitment of CIP2A–MDC1–TOPBP1 complex from spindle pole to chromosomes
Source: Nucleic Acids Res. 2023 Mar 31;51(10):4899–913. doi: 10.1093/nar/gkad213 (PMC10250218; doi:10.1093/nar/gkad213)
Supplement: gkad213_Supplemental_File [file gkad213_supplemental_file.pdf]

Supplementary Figures

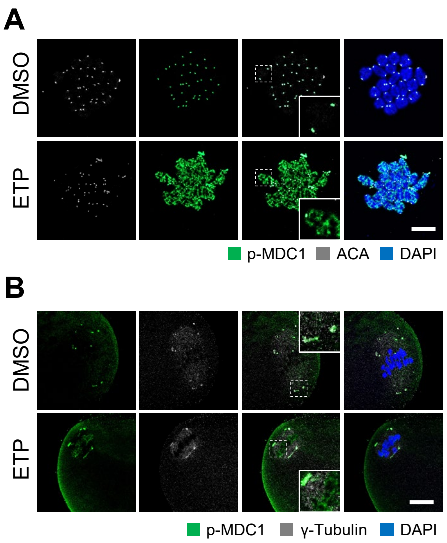

Fig. S1. Localization of p-MDC1 after DNA damage. (A) Representative images of chromosome spreads from control and ETP-treated oocytes stained with p-MDC1 and ACA antibodies. (B) Representative images of control and ETP-treated oocytes stained with p-MDC1 and γ-tubulin antibodies. Scale bar, 10 μm.

Supplementary Figures

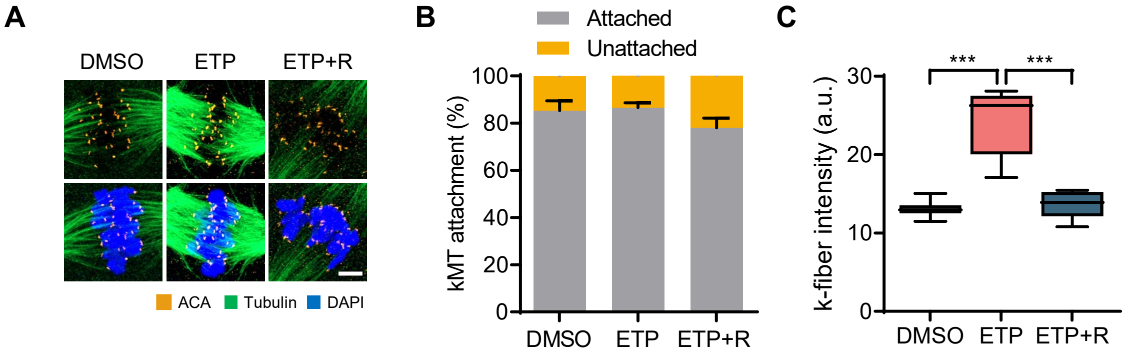

Fig. S2. Effect of DNA damage on kMT attachment. (A) Representative images of control, ETP-treated, and recovered (ETP+R) oocytes stained with tubulin and ACA antibodies after cold treatment. Scale bar, 10 µm. (B, C) Quantification of kMT attachment and k-fiber intensity. Data are presented as the mean ± SEM from three independent experiments. ***$p < 0.0001$.

Supplementary Figures

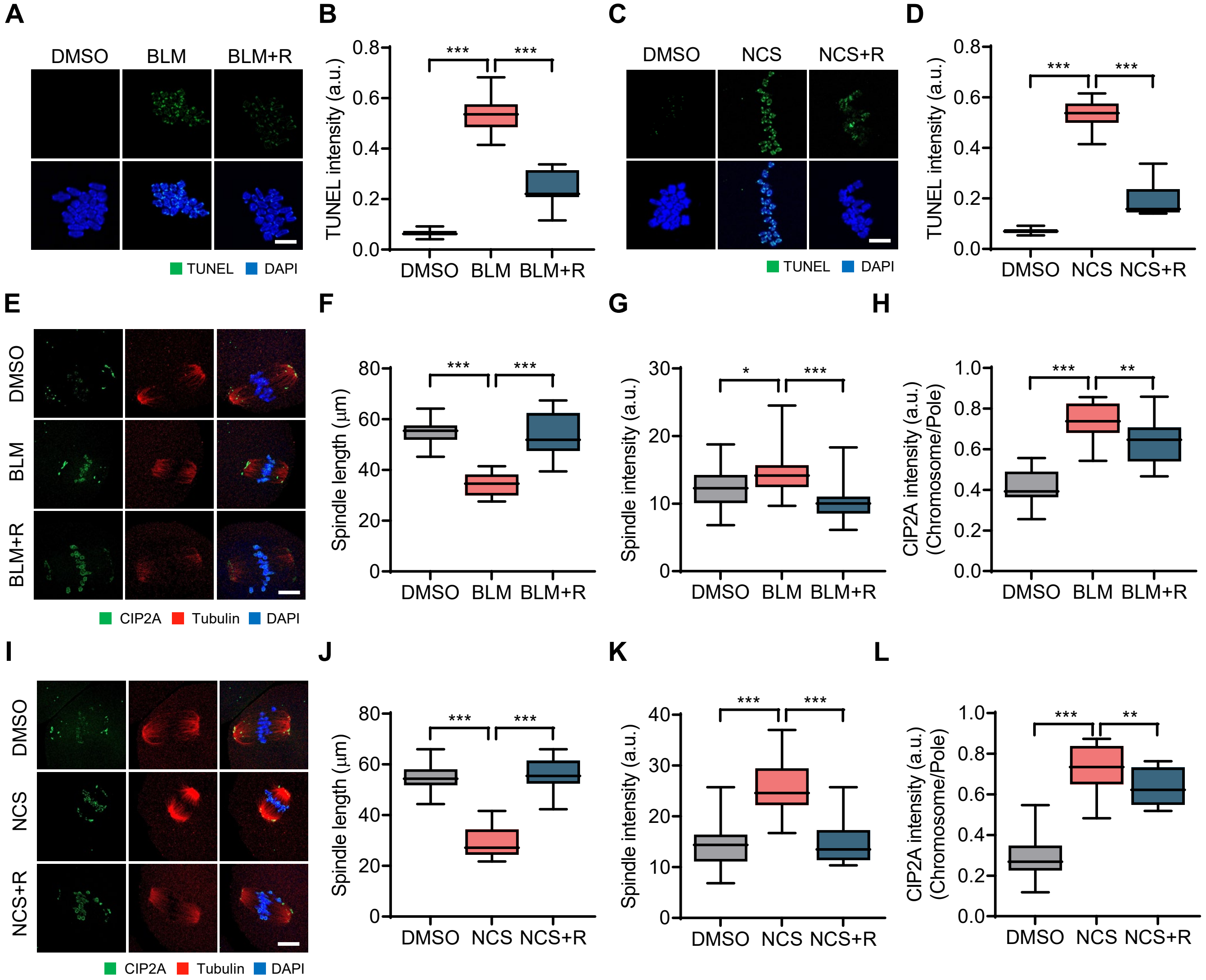

Fig. S3. DNA damage induction by bleomycin and neocarzinostatin. (A-L) Oocytes were treated with 2 μM bleomycin (BLM) or 300 nM neocarzinostatin (NCS) for 1 h and recovered from DNA damage for 2 h (BLM+R or NCS+R). Control oocytes were treated with DMSO. (A, C) Representative images of chromosome spreads showing TUNEL signals. Scale bar, 10 μm. (B, D) Quantification of TUNEL intensity. Data are presented and the mean ± SEM from two independent experiments. ***$p < 0.0001$. (E, I) Representative images showing control and BLM- or NCS-treated MI oocytes stained with CIP2A and α-tubulin. Scale bar, 10 μm. (F, G, J, K) Quantification of spindle length and intensity. Data are presented and the mean ± SEM from two independent experiments. (H, L) Ratio of CIP2A intensity at chromosomes over spindle pole. Data are presented and the mean ± SEM from two independent experiments. *$p < 0.05$, **$p < 0.001$, ***$p < 0.0001$.

Supplementary Figures

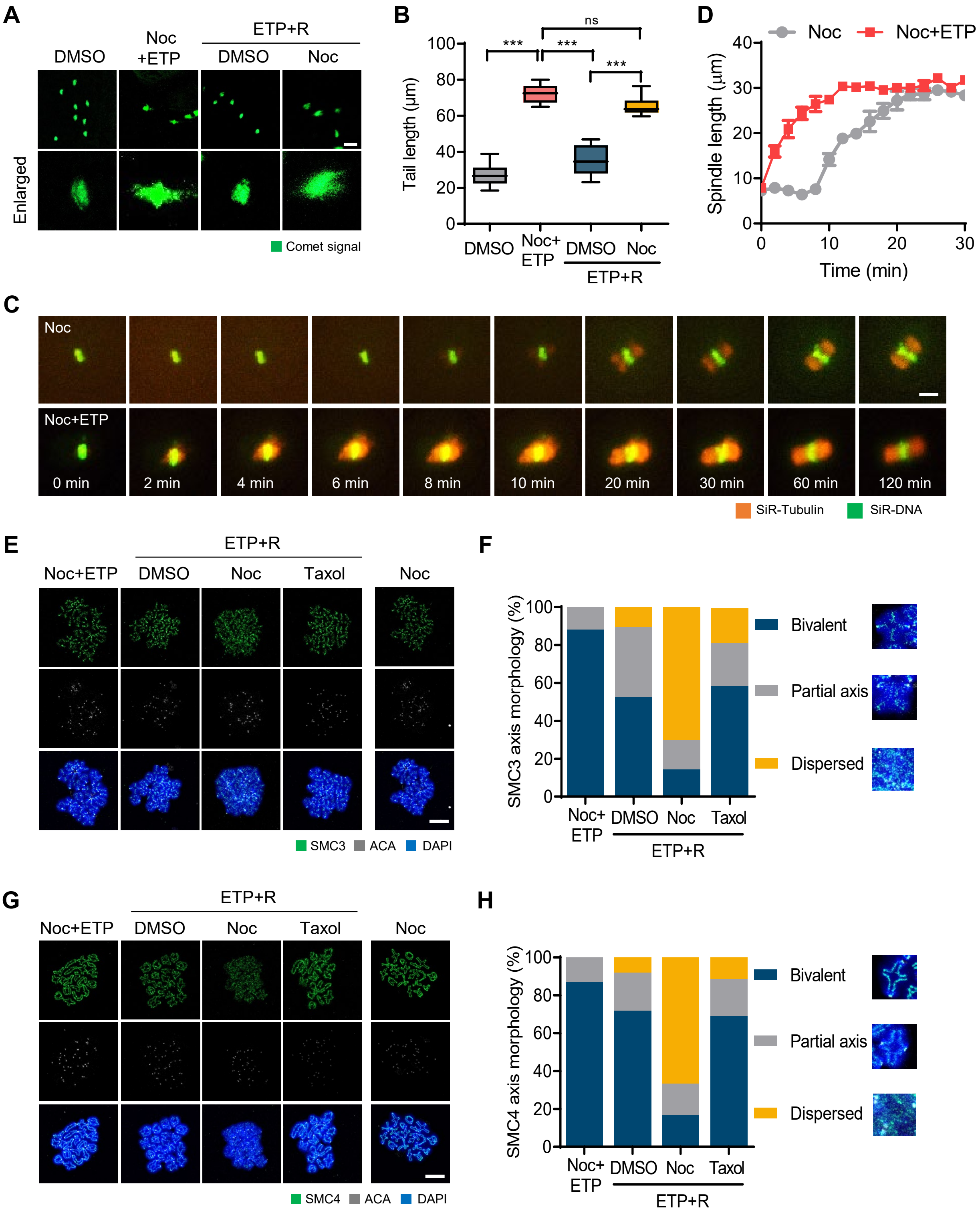

Fig. S4. Role of spindle microtubules in maintaining chromosome integrity during DSB repair. (A) Representative images showing comet signals after nocodazole (Noc) treatment during recovery. Scale bar, 50 μm. (B) Quantification of the tail length of the comet. ***$p < 0.0001$, ns, not significant. (C) Representative time-lapse images of spindles and chromosome during recovery from Noc or Noc+ETP. The time after nocodazole washout is indicated. Scale bar, 10 μm. (D) Quantification of spindle length. (E, G) Representative images of chromosome spreads stained with SMC3 or SMC4 antibodies. Scale bar, 10 μm. MI oocytes were treated with ETP with Noc for 30 min and recovered in fresh medium for 2 h with Noc or Taxol. Control oocytes were treated with Noc for 2.5 h. (F, H) Quantification of SMC3 or SMC4 axis morphology.

Supplementary Figures

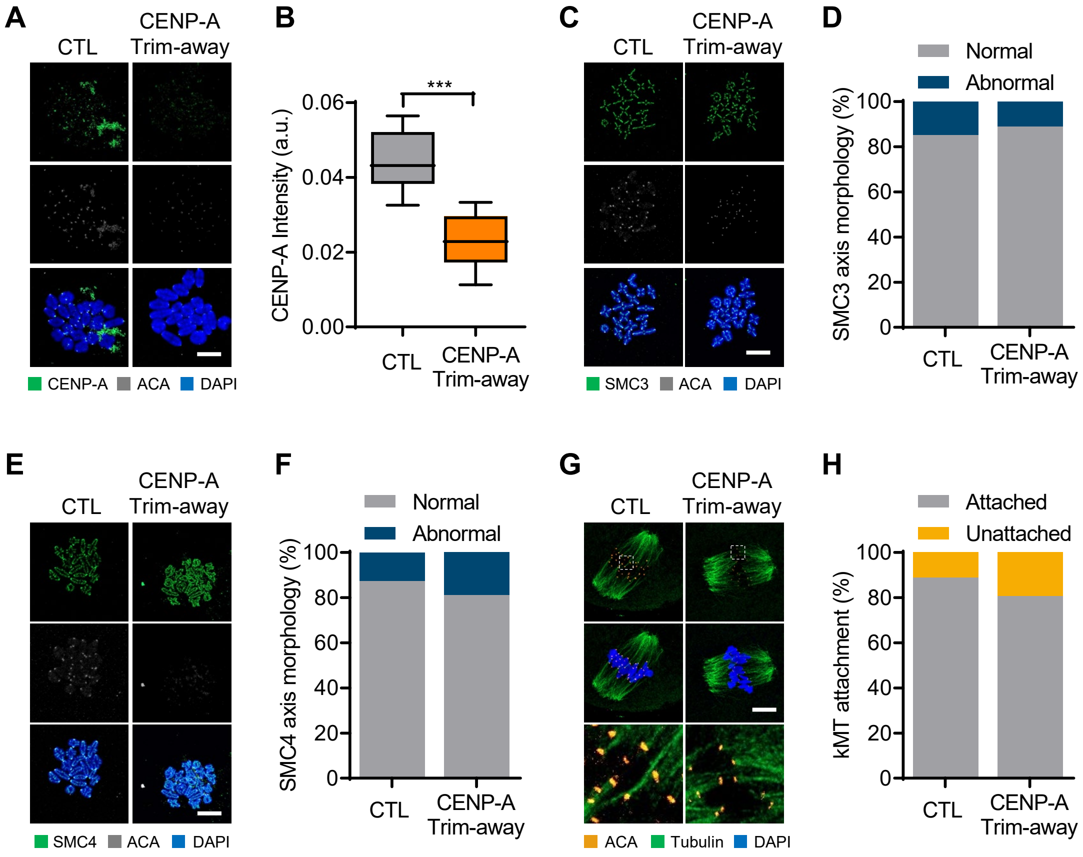

Fig. S5. Chromosome architecture after CENP-A depletion. (A, C, E) Representative images of chromosome spreads from control and CENP-A Trim-away oocytes. Scale bar, 10 μm. (B) Quantification of CENP-A intensity. Data are presented as the mean ± SEM from three independent experiments. ***p < 0.0001. (D, F). Quantification of SMC3 or SMC4 axis morphology. (G) Representative images of control and CENP-A Trim-away oocytes stained with tubulin and ACA antibodies after cold treatment. Scale bar, 10 μm. Boxed regions are enlarged in the bottom panel. (H) Quantification of kMT attachment. Data are presented as the mean ± SEM from three independent experiments.

Supplementary Figures

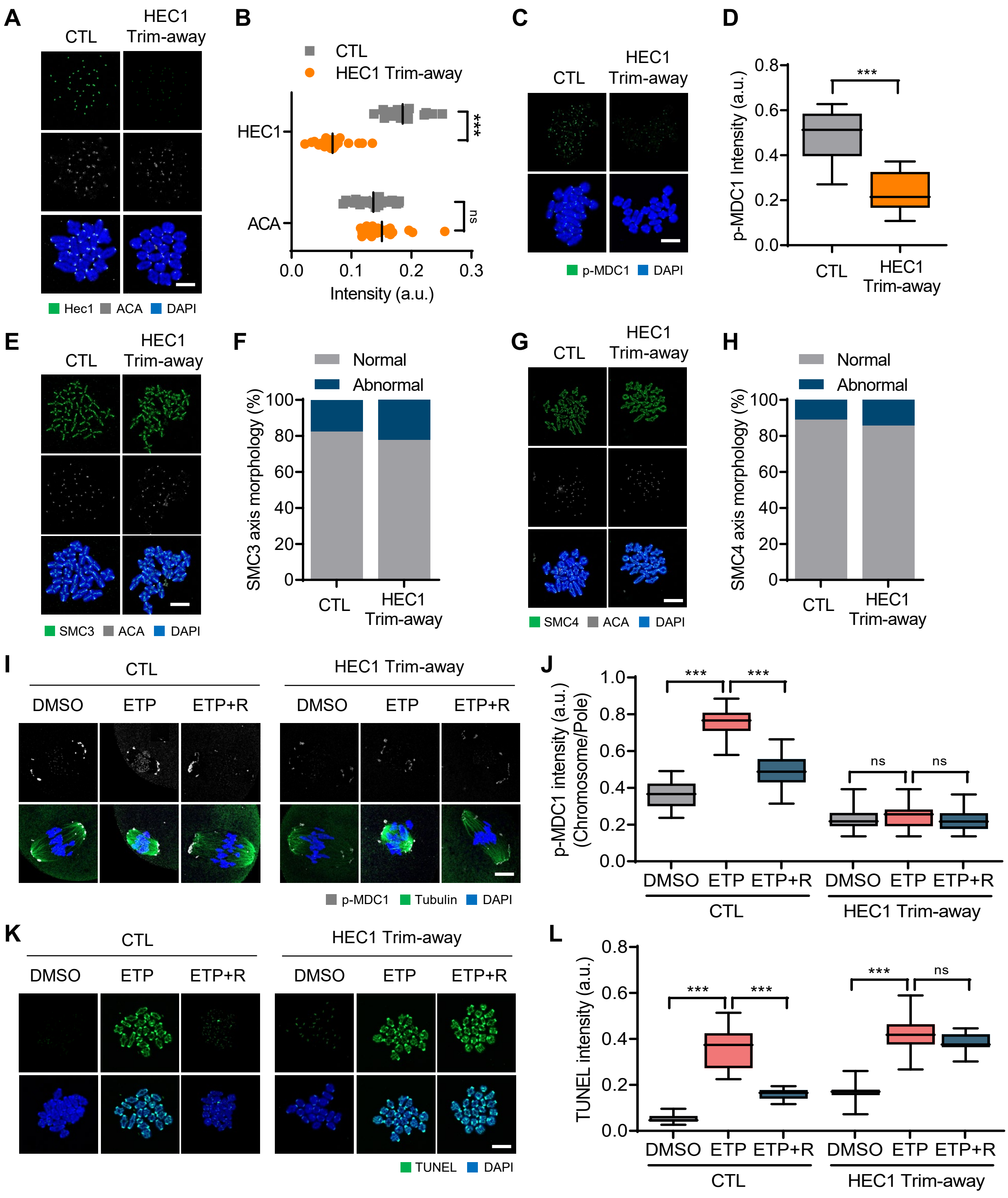

Fig. S6. Impaired relocation of CIP2A-MDC1-TOPBP1 complex after DNA damage. (A, C, E, G) Representative images of chromosome spreads from control and HEC1 Trim-away oocytes. Scale bar, 10 μm. Chromosome spreads were stained with HEC1, p-MDC1, SMC3, and SMC4 antibodies. (B, D) Quantification of HEC1, ACA and p-MDC1 intensities. Data are presented as the mean ± SEM from three independent experiments. ***p < 0.0001, ns, not significant. (F, H) Quantification of SMC3 and SMC4 axis morphology. (I) Representative images of control and HEC1 Trim-away oocytes stained with p-MDC1 and tubulin antibodies after ETP treatment and recovery (ETP+R). Scale bar, 10 μm. (J) Ratio of p-MDC1 intensities at chromosomes over spindle poles. Data are presented as the mean ± SEM from three independent experiments. ***p < 0.0001, ns, not significant. (K) Representative images of chromosome spreads showing TUNEL signals from control and HEC1 Trim-away oocytes after ETP treatment and recovery (ETP+R). Scale bar, 10 μm. (L) Quantification of TUNEL intensity. Data are presented as the mean ± SEM from three independent experiments. ***p < 0.0001, ns, not significant.

Supplementary Figures

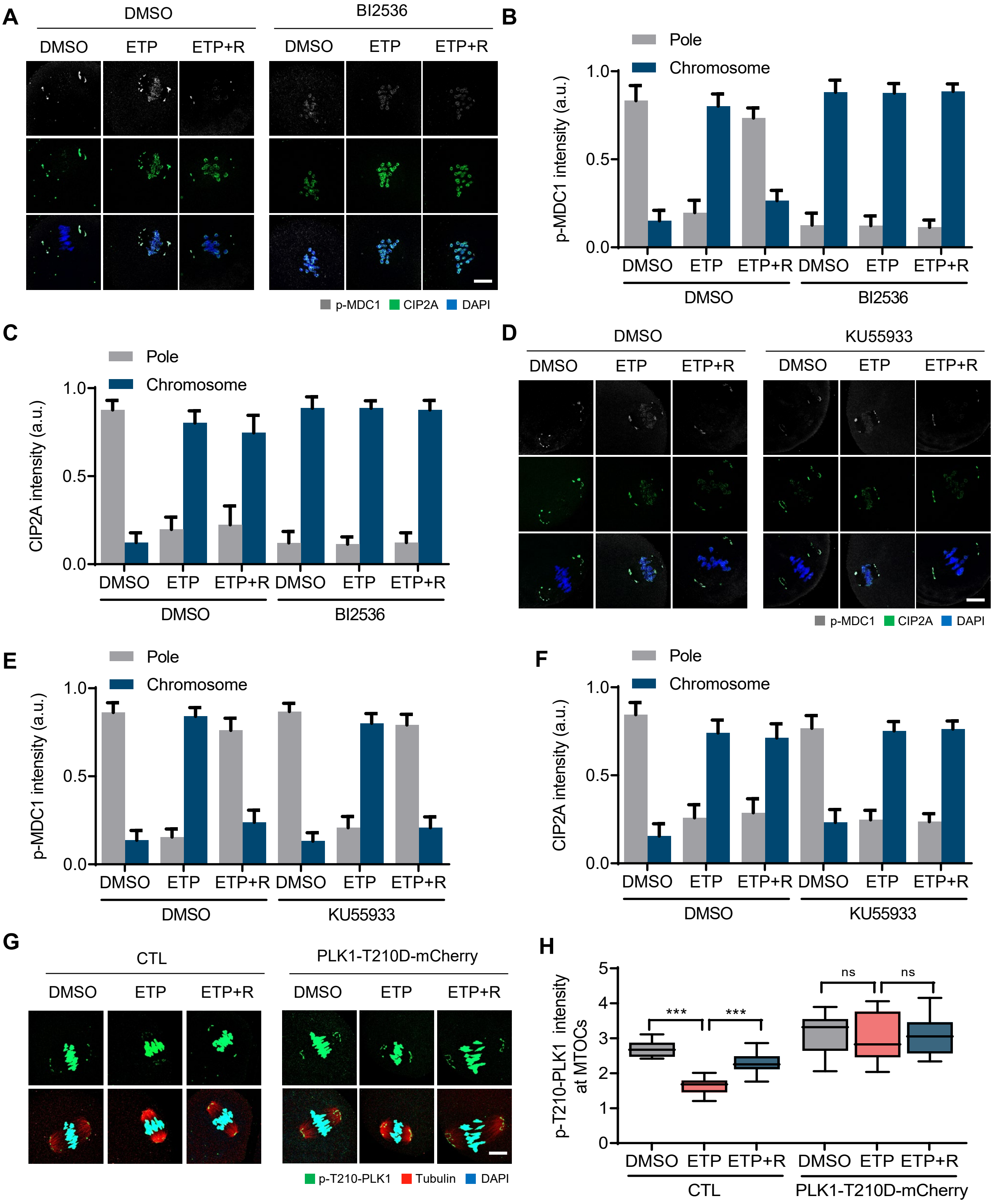

Fig. S7. PLK1-dependent relocation of CIP2A-MDC1-TOPBP1 complex. (A, D) Representative images of oocytes treated either BI2536 or KU55933 after ETP treatment and recovery (ETP+R). Control oocytes were treated with DMSO. CIP2A and p-MDC1 signals are shown. Scale bar, 10 μm. (B, C, E, F) Quantification of p-MDC1 and CIP2A localization (chromosome vs pole). Data are presented as the mean ± SEM from three independent experiments. ***p < 0.0001, ns, not significant. (G) Representative images showing p-MDC1 localization in oocytes expressing either mCherry or PLK1-T210D-mCherry after ETP treatment and recovery (ETP+R). Scale bar, 10 μm. (H) Quantification of p-T210-PLK1 intensity at MTOCs. Data are presented as the mean ± SEM from three independent experiments. ***p < 0.0001, ns, not significant.

Supplementary Figures

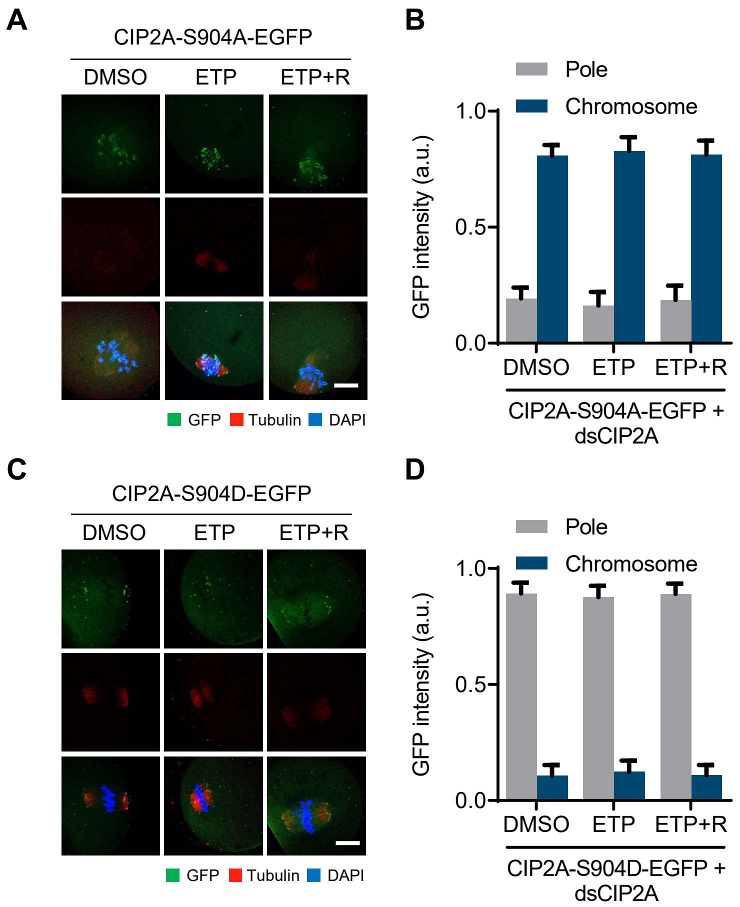

Fig. S8. Localization of CIP2A-S904A-EGFP and CIP2A-S904D-EGFP. (A, C) Representative images of oocytes overexpressing CIP2A-S904A-EGFP or CIP2A-S904D-EGFP after ETP treatment and recovery (ETP+R). MI oocytes were stained with tubulin and GFP antibodies. Scale bar, 10 μm. (B, D) Quantification of CIP2A localization (chromosomes vs pole).

Supplementary Figures

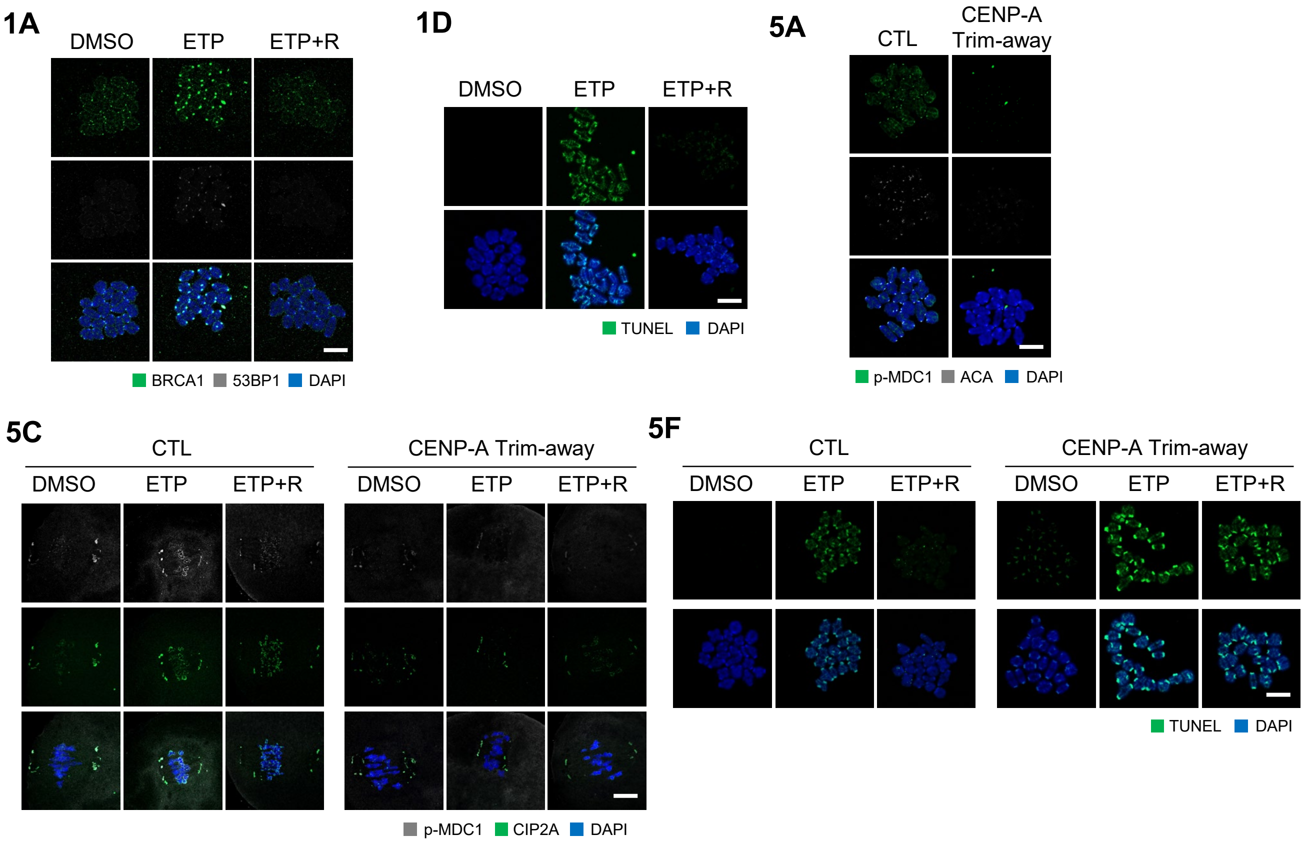

Fig. S9. Raw images of Figure 1 and Figure 5. Unprocessed raw images without background subtraction in Fig 1 and Fig 5 are shown.
